# An improved artificial gorilla troops optimizer for BP neural network-based housing price prediction

**DOI:** 10.1371/journal.pone.0332439

**Published:** 2025-09-17

**Authors:** Yulin Li

**Affiliations:** University of Birmingham Joint Institute at Jinan University, Jinan University, Jinan, China; Southwest University of Science and Technology, CHINA

## Abstract

In the context of global economic austerity in the post epidemic era, housing, as one of the basic human needs, has become particularly important for accurate prediction of house prices. BP neural network is widely used in prediction tasks, but their performance is easily affected by weights and biases, and thus metaheuristic algorithms are needed to optimize the network parameters. Firstly, to address the shortcomings of the artificial Gorilla Troops Optimizer (GTO) in such optimization tasks, such as reduced population diversity, easy to fall into local optimal solutions and slow convergence, this paper proposes a fitness allocation strategy, a Cauchy variation strategy, and an elite evolution mechanism to improve the algorithm, which in turn results in an improved artificial Gorilla Troops Optimizer (IGTO). Subsequently, a BP neural network house price prediction model based on IGTO is constructed and experiments are conducted on four datasets, namely, Boston, California-Bay, California-Land and Taiwan. The experiments are first compared with eleven other swarm intelligence algorithms and then with four machine learning models, and the results show that IGTO-BPNN improved 17.66%, 18.27%, 28.10%, 49.35% and 24.83% on five evaluation metrics, namely, MAE, MAPE, R2, RMSE, and SMAPE, respectively. The improvement of these indicators fully proves the superiority and effectiveness of IGTO-BPNN in house price prediction.

## 1. Introduction

The post epidemic era is upon us and the global economy is tightening. Housing is one of the major expenditure items for households and there is an increasing demand for house price forecasting [1]. Housing prices, a crucial economic factor, hold significant importance for governments, developers, investors, and buyers [2]. Accurate predictions are essential due to the market’s changes and volatility. The property market affects national economic growth and urbanization [3].Historically, research on housing price prediction models can be categorized into two types: econometric statistics [4] and machine learning [5]. Oladunni, Timoth et al [6] used hedonic pricing theory to investigate the real estate market and improve the pricing model based on econometric concepts to benefit both home buyers and sellers. Similarly, Ling Yuheng [7] proposed a hedonic housing model to deal with both spatial and temporal latent structural issues and conducted an empirical study on apartment transaction prices in Corsica (France). Although the econometric model is characterized by simplicity and low computational effort, the accuracy of the prediction results is poor. With the integration of computer systems and the optimization of algorithm structures, machine learning methods have provided more excellent performance for house price forecasting [8,9].

Han, Eun-Joo et al [10] used LSTM and kernel density estimation information to predict apartment prices in Seoul and conducted comparative experiments with various models such as regression analysis and deep neural networks. The experimental results show that LSTM, which can reflect the time series, is more suitable for predicting house prices. Iyad Abu Doush et al [11] used an improved moth-flame optimization algorithm to enhance the performance of the training process of Multi-Layer Perceptron Neural Network, and it was applied to the iron ore price prediction problem. Based on the characteristics of houses and the conditions of the region they are located in, Zhu, Tianlei et al [12] utilized the machine learning model XGBoost and the grid search technique to predict house prices. Considering the complex relationships among multiple features in the house price dataset, Mostofi, Fatemeh et al [13] developed a hybrid multivariate graph convolutional network (GCN) to obtain richer inputs from multi-dependent relational information. The method has good prediction accuracy compared to other machine learning methods and also provides valuable insights into the factors affecting house prices. Iyad Abu-Doush et al [14] proposed an improved Harris Hawks optimizer to regulate the control parameters of a Multi-Layer Perceptron Neural Network model and applied it to gold price prediction. The experiments fully proved the effectiveness of the model. Xu, Wei et al [15] proposed a message-passing based Bayesian learning algorithm (EM-TDAMP), which provides a large number of numerical results to prove the advantages of the method through the application of house price prediction in Boston. Although machine learning techniques have good performance in prediction tasks, they also have limitations, such as high data requirements, complex training, and large computational costs. Among various machine learning techniques, BP neural networks stand out due to their simple training process, low data requirements, high efficiency, and strong universality, and have been widely applied in prediction tasks by scholars.

In order to study the relationship between the accessibility along the urban rail transit and the change of house price, Li Ruo-qi et al [16] used a BP neural network, and the experiment proved that the mean square error of the prediction result of this method was significantly reduced. Zhao Xiaoman et al [17] established a model of the influencing factors of hedonic commercial house price, and explored the application of the BP neural network in the prediction of the price of commercial house in Hefei. Nevertheless, the BP neural network has drawbacks, including slow error convergence and a tendency to fall into finite optimal solutions. This issue arises because the BP algorithm fundamentally operates as a steepest descent method based on gradient descent [18], using the first derivative of the error with respect to weights and biases to guide subsequent adjustments direction. The initial biases and weights in this network structure are critical to its predictive performance. As a result, many domestic and international researchers have begun employing BP neural networks enhanced by swarm intelligence algorithm (SI) to address these challenges [19,20].

Wang, Jining [21] et al. combined genetic algorithm and particle swarm optimization algorithm in order to improve the BP neural network, which resulted in the creation of a GA-PSO-BP neural network model that provided accurate carbon price prediction. Wang, Yuqin [22] designed an adaptive genetic-backpropagation (BP) neural network algorithm for solving the cavitation problem of centrifugal pumps during operation. Eldeghady, Ghada Shaban [23] et al. proposed a heuristic particle swarm optimization coupled with backpropagation neural network (BPNN-PSO) technique to improve the convergence and prediction accuracy of the fault diagnostic system for photovoltaic array systems. He, Zhongying [24] used a modified back-propagation neural network model (CPSO-BP-MC) using Coordinated Particle Swarm Optimization (CPSO) algorithm and Markov Chain (MC) technique to predict the bridge condition index. This shows the effectiveness of the stochastic iterative approach using SI instead of gradient descent in optimizing the weights and biases of BP neural networks, which is widely used in several fields.

In 2021, researchers introduced a novel swarm intelligence algorithm called the Artificial Gorilla Troops Optimizer (GTO) [25]. The primary objective of this algorithm is to formalize and emulate the mass behavior exhibited by gorillas, in order to develop innovative methods for both exploration and exploitation purposes. Consequently, it quickly gained popularity among scholars and found widespread applications in various domains. Ramadan, Ashraf et al [26] used GTO algorithm to solve the multi-objective function as a way to optimize the location and rating allocation of renewable distributed generators (RDG). The algorithm resulted in significant reduction in expected cost, radiation and voltage deviation in power systems. Asaad, Ali et al [27] used GTO algorithm to optimize the allocation of renewable energy sources and electric vehicle charging stations, which effectively increased the profit of the energy company and shortened the waiting time for charging of electric vehicles. For unit commitment (UC) optimization problem, Rihan, Mahmoud [28] used GTO algorithm to solve the UC problem in three different cases, which helps to save the operational cost.

Muruga, Sathesh [29] introduced an artificial gorilla troops optimizer for artificial neural networks to manage energy consumption in hybrid power distribution networks and experimentally demonstrated that the artificial gorilla troops optimizer-enhanced neural network energy management system provides 99.55% efficiency. Konakoglu, Berkant [30] evaluated the novel hybrid artificial neural network-artificial gorilla troops optimizer (ANN-GTO) to demonstrate superior ability in predicting zenithal wet delays. Wang, Bo [31] et al. utilized gated loop circuits with an artificial gorilla troops optimizer to efficiently optimize residential energy forecasts. Lin, Hanlei [32] designed a gorilla optimization kernel extreme learning with an improved Learner’s GoogleNet Migration Learning Model (GNet TL with IGT-KELM) for accurate detection of asphalt pavement cracks. The above studies show that the GTO algorithm is successful in neural network optimization, highlighting its superior performance. However, there are fewer studies related to the optimization of BP neural networks using the GTO algorithm for the problem of house price prediction.

As mentioned earlier, the current real estate market is in a fluctuating state, and the global economy also shows a tightening trend. In this context, the BP neural network has emerged as one of the many methods with its own unique advantages. It has a strong nonlinear fitting ability, which makes it extremely suitable for application in the field of house price prediction. However, the traditional BP neural network has certain limitations, and its performance is significantly affected by the initial weights and bias. In view of this, in order to optimize the network structure, this paper adopts stochastic optimization method to replace the gradient descent method. Meanwhile, the GTO algorithm has the advantage of simple structure, and its unique exploration and development mechanism gives it excellent global optimization ability. Based on the above characteristics, this paper selects GTO algorithm as a stochastic optimization method to train BP neural networks, and then constructs the GTO-BPNN house price model. However, a large number of pre-experimental results show that the GTO-BPNN model exposes some problems in the process of house price prediction, such as easy to fall into the local optimal solution and unable to converge quickly. Therefore, in order to improve the model performance, it is necessary to improve the GTO algorithm in this paper, so as to construct a new house price prediction model IGTO-BPNN. Table 1 is an explanation of abbreviations in this paper. These main contributions are summarized as follows:

**Table 1:** Abbreviation explanation.

Abbreviation	Description	Abbreviation	Description
SI	Swarm Intelligence algorithm	FDB_TLABC	Teaching-Learning Artificial Bee Colony algorithm combined with Fitness Distance Balance [33]
SI-BPNN	BP neural network optimized by swarm intelligence algorithm	LSTM	Long Short-Term Memory
BPNN	Back Propagation Neural Network	RBF	Radial Basis Function neural network
GTO	Gorilla Troops Optimizer [25]	ELM	Extreme Learning Machine
PSO	Particle Swarm Optimization [34]	CNN	Convolutional neural network
SaDE	Self-adaptive Differential Evolution [35]	MAE	Mean Absolute Error
GWO	Grey Wolf Optimizer [36]	MAPE	Mean Absolute Percentage Error
MFO	Moth-Flame Optimization [37]	R^2^	R-square
WOA	Whale Optimization Algorithm [38]	RMSE	Root Mean Square Error
HHO	Harris Hawk Optimization [39]	SMAPE	Symmetric Mean Absolute Percentage Error
CMA-ES	Covariance Matrix Adaptation Evolutionary Strategy [40]	WSO	White Shark Optimizer [41]
EHO	Elk herd Optimizer [42]

(1) The fitness allocation strategy dynamically adjusts population positions to enhance diversity, while the Cauchy mutation operator prevents convergence to suboptimal solutions. Additionally, the survival of the fittest mechanism evolves elite individuals and eliminates weaker ones, improving the algorithm’s global search capability. The improvement of GTO algorithm by three strategies forms IGTO.
(2) The improved Artificial Gorilla Troops Optimizer (IGTO) is utilized to optimize these weights and biases of the BP neural network.
(3) Housing price prediction experiments are conducted on four datasets (Boston, California-Bay, California-Land, and Taiwan), with IGTO-BPNN compared to a BP network optimized by eleven other SI algorithms and four machine learning models (LSTM, ELM, RBF, and CNN).

**Fig 1:**
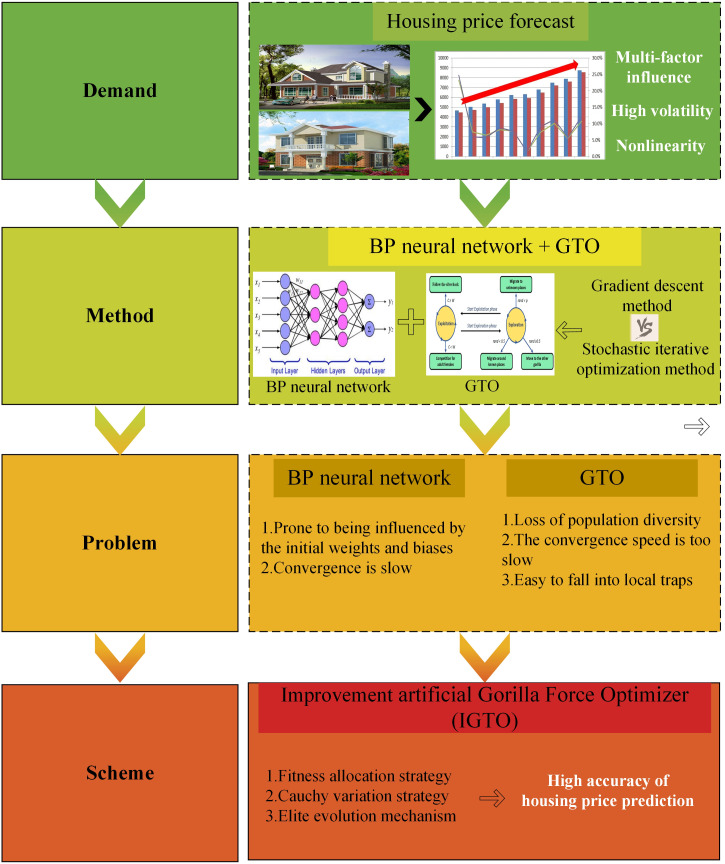
Motivation for the Paper.

Fig 1 is the motivation for the paper. The remaining part of the study is structured as follows: an overview of the BP neural network and GTO algorithm is provided in Section 2. Section 3 describes the improvement motivation and strategy of IGTO algorithm and the proposal of IGTO-BPNN model. Section 4 describes the experimental setup, including data sources, parameter settings and evaluation index. The specific trial results and related analyses are presented in Section 5. Finally, the research work and future improvement direction of the paper are summarized in Section 6.

## 2. Related works

### 2.1. Back propagation neural network

The main working principle of BP neural network is divided into two stages: forward transfer of information and back propagation of error. In the process of forward transmission, firstly, by normalizing the data, each input is connected with the weighted total score ϑ which is calculated by Eq. 1.


$$\vartheta_{b}=\sum_{a=1}^{A}(X_{a}\cdot w_{ab})-\varepsilon_{b}$$ (1)

Where *A* represents the number of input neurons. *w*_*ab*_ is the connection weight vector between the a-th input neuron and the b-th hidden neuron, and *X*_*a*_ is the a-th input neuron. *ε*_*b*_ represents the bias of the b-th hidden neuron. The intermediate vectors are then processed using the tansig activation function as shown in Eq. 2.


$$tansig(X)=\frac{\text{2}}{1+e^{-2X}}-1$$ (2)

When the information enters the output layer, it is judged whether the error between the predicted value and the true value satisfies the demand. If it is not satisfied, the error is calculated and the error back propagation stage is entered. Using the gradient descent method, the first-order derivative of the error is used to guide the updating of the weights and biases in the network structure, and then the forward propagation is continued until the error meets the demand, and the predicted value ${\hat{y}}_{c}$ is output, as shown in Eq. 3.


$${\hat{y}}_{c}=\sum_{c=1}^{C}f(X)\cdot w_{bc}+\varepsilon_{c}$$ (3)

**Fig 2:**
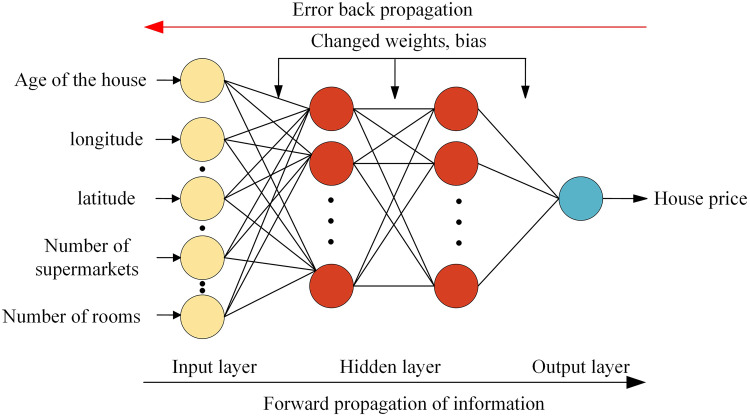
The frame of BPNN.

Where *C* is the number of neurons in the output layer, *f(X)* is the matrix of transformation vectors in the hidden layer. *w*_*bc*_ stands for the b-th hidden neuron connecting weights to the c-th output neuron, and *ε*_*c*_ refers to the bias of connecting to the c-th hidden neuron. From Eq.1 and Eq.3, it can be obtained that the weights and biases in the network structure play a key role in the performance of BP neural networks. Therefore, finding suitable and appropriate weights and biases is a crucial task because they help to enhance the performance of BP neural networks and thus improve the prediction accuracy. Fig 2 shows the frame of BPNN.

### 2.2. Artificial gorilla troops optimizer

A gorilla group, referred to as a troop, consists of a silverback gorilla, multiple adult males and females, as well as their offspring. The leader of the group is the only silverback gorilla, which directs the ‘troops’ migration, foraging and resting, while the male gorilla usually tends to migrate to another group, compete for spouse rights with other male gorilla, and then form a new group with the female gorilla. Additionally, male gorillas serve as potential candidates for assuming the role of the silverback gorilla leader. This range of natural behaviors served as inspiration for the development of GTO [25]. This algorithm has been widely used to optimize various engineering problems [43–45]. The method follows a detailed process outlined as follows:

I. Exploration Phase:


$$\zeta \left(t+1\right)=\left\{ \begin{array}{c} \left(upper-lower\right)\times \upsilon_{1}+lower,\upsilon <p\\ \left(\upsilon_{2}-\beta \right)\times \zeta_{r}\left(t\right)+A\times B,\upsilon \geq 0.5\\ \zeta \left(t\right)-A\times \left(A\times \left(\zeta \left(t\right)-\zeta_{r}\left(t\right)\right)+\upsilon_{3}\times \left(\zeta \left(t\right)-\zeta_{r}\left(t\right)\right)\right),\upsilon <0.5 \end{array}\right.$$ (4)

Eq. 4 includes several variables and parameters, such as $\upsilon_{1},\upsilon_{2},\upsilon_{3},\upsilon$,which are arbitrary numbers ranging from 0 to 1. ζ(t) denotes the position of the population at the *t-th* iteration. The maximum and minimum values of the variables are represented by *upper* and *lower*, respectively. The variable *p* takes a value within [0,1]. A randomly chosen participant, denoted as $\zeta_{r}$, is defined within the formula. Additionally, *A, B*, and *β* are determined using Eq. 5-9.


$$\beta =C\times \left(1-\frac{iteration}{Max\_iteration}\right)$$ (5)

$$C=\cos(2\times \upsilon_{4})+1$$ (6)

A=β×l (7)

B=D×ς(t) (8)

D=[−β,β] (9)

In the formula, *iteration* represents the current stage, *Max_ iteration* denotes this maximum iteration value, $\upsilon_{4}$ is an arbitrary number ranging from [0,1], and *l* is a randomly selected value within [−1, 1].

Ⅱ. Exploitation phase

(1) Migrate to unknown places: *β ≥ W*


$$\varsigma \left(t+1\right)=A\times E\times \left(\varsigma \left(t\right)-\varsigma_{sihverback}\right)+\varsigma \left(t\right)\;$$ (10)

$$E=({\left|\frac{\text{1}}{Nall}\sum_{i=1}^{Nall}\varsigma_{i}(t)\right|}^{F}{)}^{\frac{\text{1}}{F}}$$ (11)

$$F=2^{A}$$ (12)

Within Eq. 10–12, $\varsigma_{sihverback}$ is the place of the silverback gorilla, known as the optimal solution. *Nall* denotes the number of populations.

(2) Batter for mature women: *β < W*


$$\varsigma (t+1)=\varsigma_{silverback}-(\varsigma_{silverback}\times \delta -\varsigma (t)\times \delta )\times G$$ (13)

$$\delta =2\times \upsilon_{5}-1\;$$ (14)

G=κ×H (15)

$$H=\left\{ \begin{array}{c} \lambda_{1},\upsilon \geq 0.5\\ \lambda_{2},\upsilon <0.5 \end{array}\right.$$ (16)

**Fig 3:**
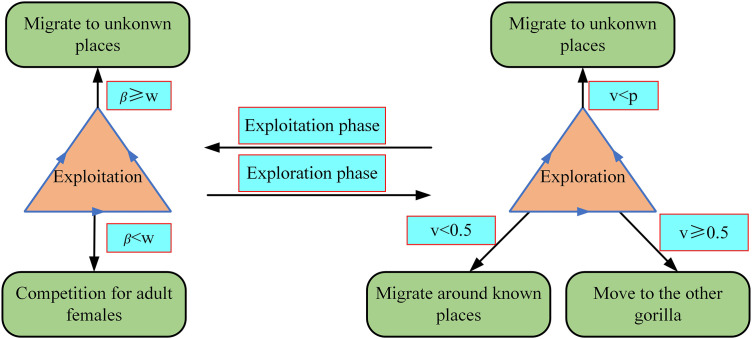
The GTO algorithm process.

Within Eq. 13–16, $\upsilon_{5}$ is arbitrary number within the range of 0–1, and *κ* is the initial preset value. If υ≥0.5, *H* represents an arbitrary number corresponding to this problem dimension and this normal distribution; otherwise, *H* is an uncertain number from the normal distribution. This GTO algorithm process is shown in Fig 3.

## 3. The proposal of IGTO-BPNN model

### 3.1. Improvement artificial gorilla troops optimizer (IGTO)

The NFL theorem [46] states that there is no comprehensively supreme algorithm that fulfills well for all challenges. The GTO algorithm is characterized by a fast search speed, wide exploration range, and good stability. Nonetheless, challenges persist when nearing the optimal solution, including diminished population diversity and convergence to finite optimal solutions. Our research team has conducted extensive scientific research on swarm intelligence optimization algorithms [47,48]. this article aims to enhance GTO from three specific aspects.

#### 3.1.1. Fitness allocation strategy.

In a gorilla population, each male with superior physical attributes and posture tends to attract more females, thereby gaining mating rights. This behavior, known as “fitness allocation”, is modeled mathematically to simulate the dynamic adjustment of fitness weights assigned to individuals during each iteration. These weights are continuously adjusted throughout the optimization process to enhance the effectiveness of finding the optimal solution. The formula is as follows.


$$\zeta \left(t+1\right)=\left\{ \begin{array}{c} \left(upper-lower\right)\times \upsilon_{1}+lower,\upsilon <p\\ \zeta (t)\times levy+\frac{Finess_{\zeta (t)}-WROST_{\zeta (t)}}{BEST_{\zeta (t)}-WROST_{\zeta (t)}},\upsilon \geq 0.5\\ \zeta \left(t\right)-A\times \left(A\times \left(\zeta \left(t\right)-\zeta_{r}\left(t\right)\right)+\upsilon_{3}\times \left(\zeta \left(t\right)-\zeta_{r}\left(t\right)\right)\right),\upsilon <0.5 \end{array}\right.$$ (17)

In Eq. 17, *levy* refers to Levy flight strategy. The fitness function value of the individual $Finess_{\zeta (t)}$ is calculated at the *t-th* iteration, with the best and worst fitness values denoted as $BEST_{\zeta (t)}$ and $WROST_{\zeta (t)}$, respectively.

#### 3.1.2. Cauchy variation strategy.

The Cauchy distribution is characterized by its probability density function in one dimension. It is displayed in Eq. 18.


$$f(x)=\frac{\text{1}}{\pi }\times \frac{a}{a+x^{2}},x\in (-\infty ,+\infty )$$ (18)

When *a* = 1, the distribution is referred to as the standard Cauchy distribution, and it follows the generating function for random variables in this distribution, as shown in Eq. 19.


$$cauchy_{x}=\tan((x-\frac{\text{1}}{\text{2}})\times \pi )$$ (19)

Based on this, the introduction of the Cauchy mutation operator is displayed in Eq. 20 [49,50].


cauchy=1+tan(0.5×π×(rand−0.5)) (20)

The development phase of GTO is optimized in Eq. 21, where $\varsigma_{c1}(t),\varsigma_{c2}(t)$ are two different individuals randomly selected in the previous iteration process.


$$\varsigma (t+1)=\varsigma (t)+cauchy\times (\varsigma_{c1}(t)-\varsigma_{c2}(t))$$ (21)

#### 3.1.3. Elite evolution mechanism.

The law of nature is always ‘natural selection, survival of the fittest’. Gorilla in foraging, migration and other group activities, if there are members foraging, hunting ability is poor, who can’t hunt for food for the population or lead to slow migration speed, such members will become this silverback gorilla to survive and eliminate this gorilla. Assuming that this gorilla elimination proportion coefficient is Q, the population elite evolution and the last elimination mechanism will be carried out according to EQ. *round* is a rounding function.


EQ=round(Nall×Q) (22)

$$\varsigma_{new}=(upper-lower)\times \varsigma (1:EQ)+lower$$ (23)

$$\varsigma (t+1)=[\varsigma (t+1)\cup \varsigma_{new}]$$ (24)

This fitness function value for all individuals is calculated using Eq. 22-24, and the top *Nall* individuals are selected in ascending order. If solving a maximization problem, the order is reversed.

**Fig 4:**
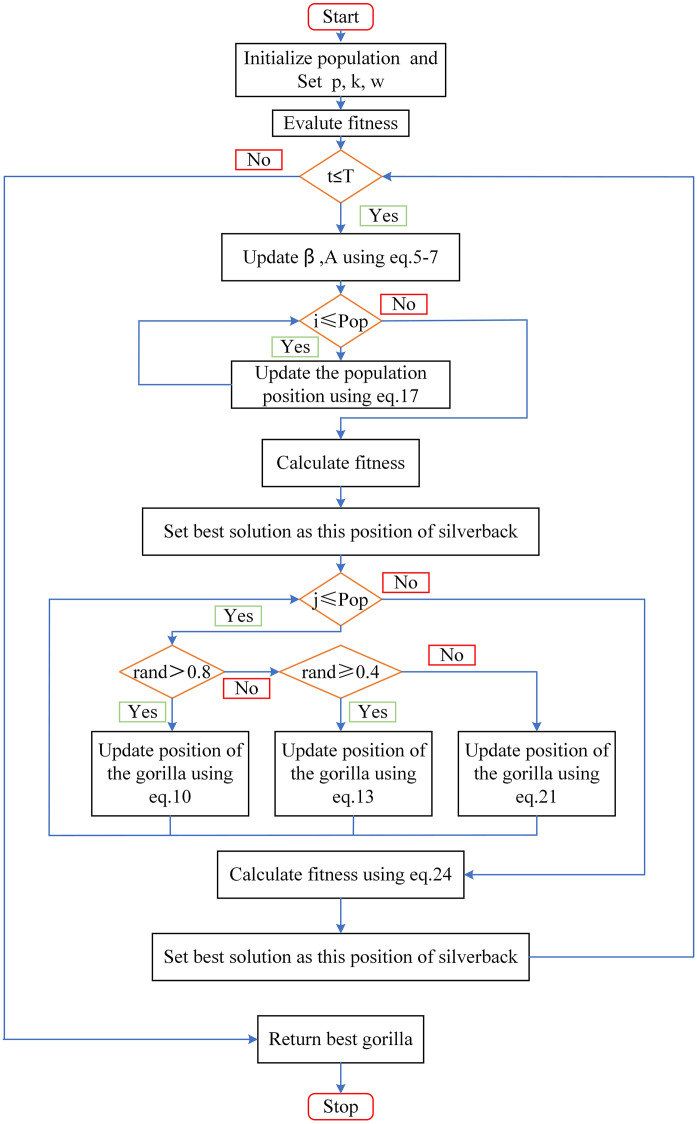
The flow chart of IGTO.

In summary, the flowchart of IGTO is shown in Fig 4, and the corresponding pseudo-code s presented in algorithm 1.

Algorithm 1 Pseudo code for IGTO

*BEGINING*

*Inputs:* ***Nall, Max_ iteration, p, κ, w***

*Initialization the individuals* 𝜍_***i***_ *using Eq. (17)*

*Calculate the fitness value*

*WHILE* ***iteration ≤Max_ iteration*** *do*

* Adjust the* ***β*** *and* ***A*** *using Eq. (5–7)*

*For (i* ***= 1,2……Nall*** *) do*

*Adjust this place of Gorilla using Eq. (17)*

*End for*

* Set* 𝜍_***silverback***_ *as this place of silverback*

*For (i* ***= 1,2……Nall*** *) do*

*  If*𝜈* > 0.8*

*Adjust the place of Gorilla using Eq. (10)*

*  Elseif 0.4 < *𝜈* ≤ 0.8*

*Adjust the place of Gorilla using Eq. (13)*

*Else*

*Adjust the place of Gorilla using Eq. (21)*

*End for*

*Calculate the fitness value using Eq. (24)*

*  Set* 𝜍_***silverback***_ *as this place of silverback*

*End while*

* Return* 𝜍_***silverback***_ *and* ***best score***

*End*

### 3.2. IGTO optimizes BP neural networks

The preliminary weights and biases of BPNN are produced at random, significantly influencing the network’s performance. The IGTO method is used to optimize the BP neural network in this paper. The step-by-step implementation process is outlined as follows:

1) Set the relevant parameters and define the network architecture of the BPNN.
2) Apply the initial individuals place of IGTO as the biases and weights of BPNN.
3) Train this neural network, optimizing it with utilizing the training error value as the fitness function of IGTO.
4) Update the positions of the individuals recursively using the IGTO algorithm to seek the best solution.
5) Check the termination condition of the method. If satisfied, terminate this optimization and output the optimal weights and biases found by IGTO. Otherwise, continue the iteration.
6) Use the best weight and bias from the output as the original weight and bias for the IGTO-BPNN model.

## 4. Experiment preparation

All experiments in this paper are carried out under Windows 11, X-64 operating system, MATLAB version is R2023a. In order to better evaluate the performance of the IGTO-BPNN house price prediction model, it is firstly compared like-for-like with BPNNs optimized by eleven SI algorithms, including: PSO [34], CMA-ES [40], SaDE [35], GWO [36], MFO [37], WOA [38], HHO [39], GTO, FDB_TLABC [33], WSO [41], EHO [42]. Then, after the comparison of the IGTO-BPNN with four classical machine learning models including: CNN [51], ELM [52], LSTM [53], RBF [54].

### 4.1. Dataset

This paper utilizes the IGTO-BPNN model to predict house prices. A total of four public data sets are taken, which are:

1) The Boston House Price Dataset [55] is hosted on the UCI platform. This data set has 14 attributes, of which the first 13 columns are used as feature inputs, including: crime rate, residential area, average number of rooms, etc. The last column is the label of the real value of the house price to be predicted, and the data set is named “Boston”.
2) California housing price data set [56], including a total of 10 columns of longitude, latitude, residential years, room total, etc., of which columns 9 and 10 are the real value label of housing price and the location area of the house (near the bay, 1 nautical mile from the sea, inland, islands). To account for the influence of independent variables, in this paper, this dataset is extracted into two datasets “close to the bay” and “inland”, each of which retains the first 8 columns as feature input and the 9th column as house price output, and the datasets are named “Ca-Bay” and “Ca-Land”, respectively.
3) Xindian District, New Taipei City, Taiwan [57], which includes a total of 7 columns such as the age of the house, nearest subway station, convenience store, etc. However, since the first column is a time series and not relevant to this article, it is removed. Consequently, the processed dataset consists of 6 columns. The last column represents the real value label of the house price and is named “Taiwan”.

### 4.2. Evaluation indicators

This paper focuses on data regression prediction and utilizes various evaluation indexes [58–60], involving Root Mean Square Error (RMSE), R-square (R^2^), Mean Absolute Percentage Error (MAPE), Mean Absolute Error (MAE) and Symmetric Mean Absolute Percentage Error (SMAPE). The calculation formula for these indexes is presented in Table 2. Generally, smaller values for these indicators signify better performance, except for R^2^, where values closer to 1 indicate better performance. In Table 2, *A* represents the number of data, $\theta_{a},\theta_{a}^{*}$ representing the true and predicted values, respectively.

**Table 2:** Calculation formula of evaluation index.

Evaluation index	Calculation formula
MAE	$MAE=\frac{\text{1}}{A}\sum_{a=1}^{A}\left|\theta_{a}-\theta_{a}^{*}\right|$
MAPE	$MAPE=\frac{\text{1}}{A}\sum_{a=1}^{A}\left|\frac{\theta_{a}-\theta_{a}^{*}}{\theta_{a}}\right|\times 100\%$
R^2^	$R^{2}=1-\frac{\sum_{a=1}^{A}(\theta_{a}-\theta_{a}^{*}{)}^{2}}{\sum_{a=1}^{A}(\theta_{a}-\overset{―}{\theta_{a}}{)}^{2}}$
RMSE	$RMSE=\sqrt{\frac{\text{1}}{A}\sum_{a=1}^{A}{\left(\theta_{a}-\theta_{a}^{*}\right)}^{2}}$
SMAPE	$SMAPE=\frac{\text{1}}{A}\sum_{a=1}^{A}\frac{\left|\theta_{a}-\theta_{a}^{*}\right|}{(\left|\theta_{a}\right|+\left|\theta_{a}^{*}\right|)/2}\times 100\%$

### 4.3. Fitness function design

In this study, the BPNN is enhanced using a SI algorithm. The mean squared error between the actual and predicted values in the training set is calculated and summed. As a result, these dynamic convergence curves of the fitness value for each SI algorithm are generated. The objective function is established as shown in Eq. 25, where *A* represents the number of data, while *X*_*real*_ and *X*_*pred*_ are the true and predicted values, respectively.


$$fitness=\sum_{a=1}^{A}\sqrt{\frac{\text{1}}{A}{\left(X_{real}-X_{pred}\right)}^{2}}$$ (25)

### 4.4. Experimental parameter setting

The initial framework of the BP neural network is constructed based on the BP theorem, which posits that a 3-layer BP neural network is sufficient to approximate any nonlinear fitting function. Considering the different data feature inputs, a 3-layer network with 6 hidden neurons is utilized in the experiment. This tansig activation function is applied to transmit signals from this input layer to this hidden layer, while this purelin activation function is employed to transfer signals from this hidden layer to this output layer. Each iteration consists of 200 trials with a learning rate of 0.01, aiming to achieve an error accuracy of 10^−6^ after training. The IGTO algorithm adopts *Nall* = 30, *Max_iteration* = 100, *p *= 0.03, *κ* = 3, and *w* = 0.8. The parameter settings for the remaining comparison experiments are provided in Table 3. All datasets are divided into training and testing sets with a 7:3 ratio. After 30 experimental cycles, the average values of all evaluation indexes are presented.

**Table 3:** Comparison experiment parameter setting.

Model	Parameter setting	Model	Parameter setting
PSO-BPNN	V_max_ = 6, W_max_ = 0.9 W_min_ = 0.6,c1 = c2 = 2	FDB_TLABC-BPNN	limit = 200, cr = 0.5
CMA-ES-BPNN	NONE	WSO-BPNN	Tau = 4.11, Fmax = 0.75, Fmin = 0.07
SaDE-BPNN	r1 = 0.5, ns1 = ns2 = nf1 = nf2 = 0	EHO-BPNN	NONE
GWO-BPNN	NONE	LSTM	MaxEpochs = 1200, InitialLearnRate = 0.01, LearnRateDropFactor = 0.5
MFO-BPNN	NONE	ELM	num_hiddens = 50, activate_model = ‘sig’
WOA-BPNN	NONE	RBF	Expansion speed of Radial basis function = 100
HHO-BPNN	NONE	CNN	MaxEpochs = 1200, InitialLearnRate = 0.01, LearnRateDropFactor = 0.1
GTO-BPNN	*p* = 0.03, *κ* = 3, *w* = 0.8		

### 4.5. Parameter sensitivity analysis

**Fig 5:**
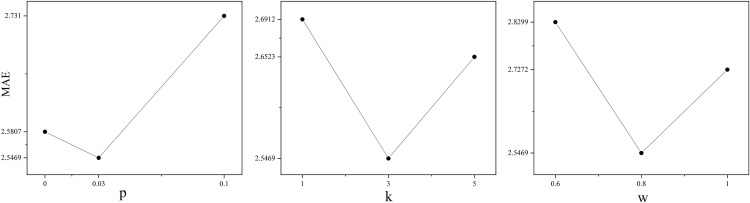
Orthogonal experimental trend diagram.

In the original GTO algorithm, the parameters are set as shown in Table 3, and the IGTO algorithm proposed in this paper does not change these parameters. The *p* in Eq.4 is used to control how the population searches in the solution set space. *k* is the set value given in Eq.15 to calculate the position of the population localized exploitation. *w* is used to control how the population chooses the position update during localized exploitation. In this subsection, the Boston house price dataset is used as an example, the number of populations is set to 30, and the maximum number of iterations is 100. For the parameter settings of the IGTO algorithm, the three parameters are respectively increased and decreased in the experiments (see Table 4). By comparing the MAE, as shown in Fig 5, it is shown that the optimal parameter settings in the IGTO algorithm are *p* = 0.03, *κ* = 3, *w* = 0.8.

**Table 4:** Different parameter Settings for IGTO.

	*p*	*k*	*w*	MAE	
1	0.03	3	0.8	2.5469	Original Settings
2	0	3	0.8	2.5807	*p*↓
3	0.1	3	0.8	2.7310	*p*↑
4	0.03	1	0.8	2.6912	*k*↓
5	0.03	5	0.8	2.6523	*k*↑
6	0.03	3	1	2.7272	*w*↑
7	0.03	3	0.6	2.8299	*w*↓

## 5. Experimental results and analysis

### 5.1. Compared with other SI-BPNN

**Fig 6:**
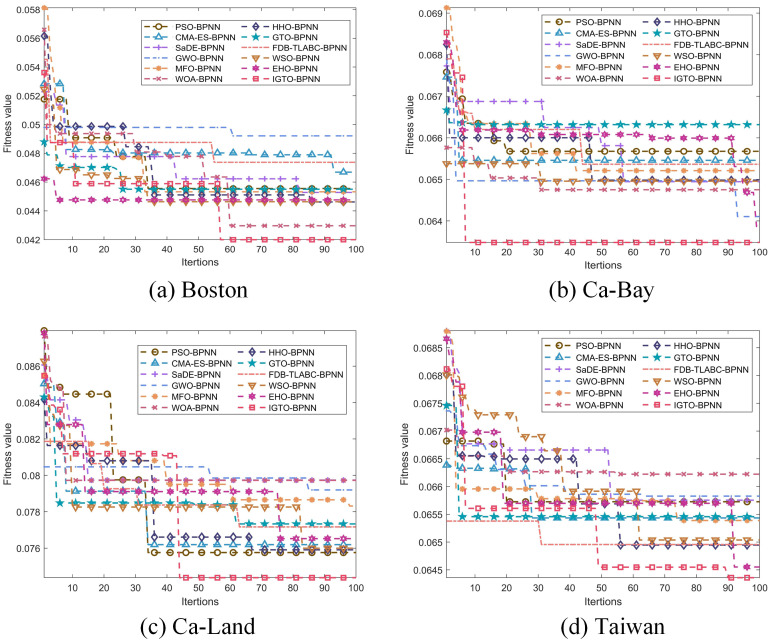
Dynamic convergence curves.

In this study, the performance of IGTO-BPNN is assessed by comparing with BPNNs optimized by eleven SI algorithms, including: PSO, CMA-ES, SaDE, GWO, MFO, WOA, HHO, GTO, FDB_TLABC, WSO, EHO. Fig 6 illustrates the dynamic convergence process of the fitness function for these twelve optimization models as they search for these best biases and weights of BPNN.

From the performance of the convergence curves, the optimization effect of IGTO-BPNN on the four house price datasets is significantly better than that of other SI algorithms. IGTO-BPNN not only performs faster in terms of convergence speed and is able to reach the stable state earlier, but also has a lower value of final fitness, which indicates that it is able to find a better solution in the optimization process. In addition, the curve of IGTO-BPNN is smoother and more stable, avoiding the fluctuations and falling into local optimal solutions that are common in other algorithms. This is due to the improvement strategies of IGTO algorithm, including the fitness allocation strategy, the Cauchy variation strategy and the elite evolution mechanism, which make IGTO perform better in global search and avoiding local optimums, thus improving the optimization efficiency and accuracy of the results. Specific data for the five evaluation metrics are tabulated in Tables 5–8-8, while standard deviations (SD) and run times are also recorded. Bold indicates the optimal value. The SD is calculated from the MAE after 30 cycles and is used to measure the stability of the algorithm.

From Tables 5–8 IGTO-BPNN shows obvious superiority in all five evaluation metrics (MAE, MAPE, R^2^, RMSE, SMAPE). Whether on Boston, Ca-Bay, Ca-Land or Taiwan datasets, the MAPE and RMSE values of IGTO-BPNN are always lower than those of other SI algorithms, showing its advantages in prediction accuracy and error control. Especially on the R^2^ value, IGTO-BPNN is able to provide a high degree of fit, especially on the Boston dataset, with an R^2^ value of 0.8881, which is much higher than that of other SI algorithms, indicating that it is more capable of capturing the data changes. Meanwhile, the MAE and SMAPE values of IGTO-BPNN are always the lowest on the four datasets, which further proves that it has less error and better stability in the optimization process. In summary, IGTO-BPNN shows higher accuracy and stability in weight and bias optimization, proving its superiority in BP neural network optimization. For the SD, IGTO – BPNN achieves the best value twice in the four datasets. In Ca-Bay and Taiwan datasets, this paper’s method did not obtain the best value, but the SD is always at a low level and tops the list. This indicates that the IGTO-BPNN model is more stable in house price prediction problem with low probability of randomness. In addition, the statistical running time is used to measure the computational efficiency of different models. The running time of the IGTO-BPNN in the Boston and Ca-Land datasets is 83.4011s and 72.6272s, respectively, which is in the first place, much better than the original GTO-BPNN model’s 113.2486s and 174.4155s, which highlights the three improved strategies in this paper. The IGTO-BPNN also ensures better computational efficiency in Ca-Bay and Taiwan datasets.

**Table 5:** The indexes of SI-BP on Boston.

Models	MAE	MAPE	R^2^	RMSE	SMAPE	SD	Time (s)
BP	2.6733	0.1516	0.6933	6.3225	0.1474	4.4883	93.4250
PSO-BPNN	2.7930	0.1432	0.7673	3.9998	0.1320	3.8299	111.4529
CMA-ES-BPNN	2.6825	0.1159	0.7705	4.2562	0.1164	4.2356	90.0198
SaDE-BPNN	2.6592	0.1391	0.7632	4.7726	0.1327	6.2432	96.7584
GWO-BPNN	2.6158	0.1239	0.8375	5.2865	0.1241	4.8208	124.5896
MFO-BPNN	2.5118	0.1219	0.8528	4.3353	0.1226	3.4113	96.1578
WOA-BPNN	2.9659	0.1411	0.7344	5.1931	0.1332	5.9418	96.5431
HHO-BPNN	2.8401	0.1442	0.7943	5.1070	0.1455	4.2619	154.9652
GTO-BPNN	2.3939	0.1167	0.8456	3.7091	0.1232	3.4356	113.2486
FDB_TLABC-BPNN	2.9346	0.1410	0.8168	5.4283	0.1364	4.2915	137.8459
WSO-BPNN	2.9152	0.1476	0.7802	4.4927	0.1402	4.4707	88.1527
EHO-BPNN	2.5841	0.1202	0.8000	4.0961	0.1166	4.0917	108.9120
IGTO-BPNN	**2.2013**	**0.1145**	**0.8881**	**3.2023**	**0.1108**	**3.3624**	**83.4011**

**Table 6:** The indexes of SI-BP on Ca-Bay.

Models	MAE	MAPE	R^2^	RMSE	SMAPE	SD	Time (s)
BP	3.5850	0.1997	0.5028	4.8310	0.1851	5.2988	91.0726
PSO-BPNN	3.3122	0.1833	0.6504	5.6617	0.1724	4.4768	83.0003
CMA-ES-BPNN	3.0492	0.1658	0.7100	4.5224	0.1609	4.5228	**52.3655**
SaDE-BPNN	3.2139	0.1700	0.7745	4.5890	0.1700	4.6000	104.6613
GWO-BPNN	3.1506	0.1787	0.6895	4.5187	0.1688	4.2236	174.6082
MFO-BPNN	3.2059	0.1806	0.6900	4.7032	0.1715	4.7043	90.0903
WOA-BPNN	3.1323	0.1876	0.6625	4.5652	0.1715	4.2052	86.4943
HHO-BPNN	3.0928	0.1621	0.6895	5.1705	0.1727	**4.0001**	87.3901
GTO-BPNN	3.1126	0.1724	0.7894	3.3580	0.1572	4.5657	86.7410
FDB_TLABC-BPNN	3.1037	0.1751	0.7346	5.9598	0.1668	4.3565	86.1038
WSO-BPNN	3.3840	0.1933	0.7121	4.8711	0.1758	4.8721	205.2954
EHO-BPNN	3.2132	0.1812	0.7305	4.5224	0.1659	4.5140	96.9358
IGTO-BPNN	**3.0292**	**0.1512**	**0.7958**	**3.3384**	**0.1514**	4.0536	83.5672

**Table 7:** The indexes of SI-BP on Ca-Land.

Models	MAE	MAPE	R^2^	RMSE	SMAPE	SD	Time (s)
BP	3.3393	0.2113	0.6592	10.4686	0.2017	7.6602	100.2453
PSO-BPNN	3.3746	0.2073	0.7374	6.8255	0.1972	4.1610	80.6591
CMA-ES-BPNN	3.1734	0.2114	0.7880	4.6388	0.1956	4.6213	82.9952
SaDE-BPNN	3.1921	0.1828	0.7266	5.2463	0.1855	4.5728	84.3534
GWO-BPNN	3.3249	0.2072	0.7688	5.7731	0.1939	6.6257	84.2584
MFO-BPNN	3.2146	0.1864	0.7681	9.1724	0.1873	8.3923	82.5143
WOA-BPNN	3.2661	0.1913	0.7668	4.9073	0.1927	6.4742	78.7084
HHO-BPNN	3.1478	0.1952	0.7464	4.5178	0.1855	4.4073	188.6136
GTO-BPNN	3.1488	0.1852	0.7881	4.2984	0.1851	6.8010	174.4155
FDB_TLABC-BPNN	3.1587	0.1920	0.7219	4.8118	0.1920	7.3468	171.5690
WSO-BPNN	**3.0366**	0.1897	**0.8272**	4.2382	**0.1830**	4.2277	89.1572
EHO-BPNN	3.2151	0.1928	0.7882	4.6764	0.1870	4.6727	96.7305
IGTO-BPNN	3.0759	**0.1760**	0.8075	**3.3879**	0.1848	**3.9757**	**72.6272**

**Table 8:** The indexes of SI-BP on Taiwan.

Models	MAE	MAPE	R^2^	RMSE	SMAPE	SD	Time (s)
BP	6.5373	0.1998	0.4357	24.3248	0.1813	10.8703	87.9582
PSO-BPNN	5.8698	0.2010	0.5904	21.8724	0.1645	8.3923	92.0108
CMA-ES-BPNN	6.2076	0.1950	0.5002	8.7391	0.1621	8.7027	86.8703
SaDE-BPNN	5.9534	0.1655	0.5800	10.4259	0.1655	7.5294	96.8215
GWO-BPNN	6.1346	0.1595	0.4933	16.4227	0.1649	7.8778	102.2088
MFO-BPNN	5.8508	0.2035	0.5982	11.8022	0.1640	10.5267	109.6815
WOA-BPNN	5.8429	0.2021	0.5922	22.4347	0.1636	8.7393	116.1754
HHO-BPNN	6.1622	0.1606	0.4933	16.3666	0.1644	7.7992	143.0606
GTO-BPNN	5.8561	0.1511	0.5996	7.0243	0.1509	8.0048	124.4274
FDB_TLABC-BPNN	5.8234	0.1639	0.6001	8.2536	0.1639	8.2830	123.8513
WSO-BPNN	6.1551	0.1527	0.5075	10.7086	0.1525	10.6645	**58.8395**
EHO-BPNN	**5.1640**	0.1766	**0.7115**	7.1591	0.1471	**7.0604**	69.5739
IGTO-BPNN	5.2275	**0.1434**	0.6583	**6.2697**	**0.1393**	7.3309	75.1857

**Fig 7:**
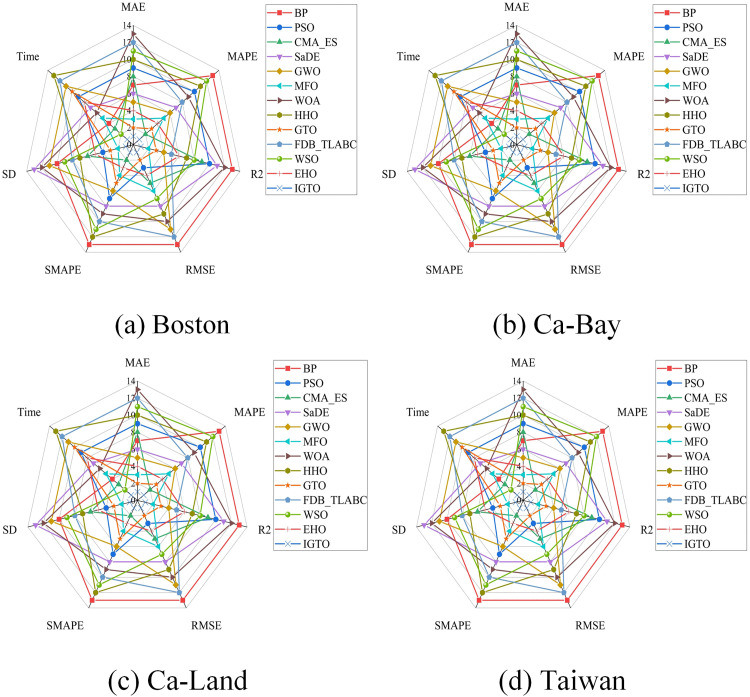
Ranking chart of SI-BPNN evaluation metrics.

Fig 7 presents the rankings of the above 13 models in the four datasets based on five evaluation metrics, which also cover SD and running time. The IGTO-BPNN model achieves a total of 19 first places, while the original GTO-BPNN model only achieves 7 second places.

**Fig 8:**
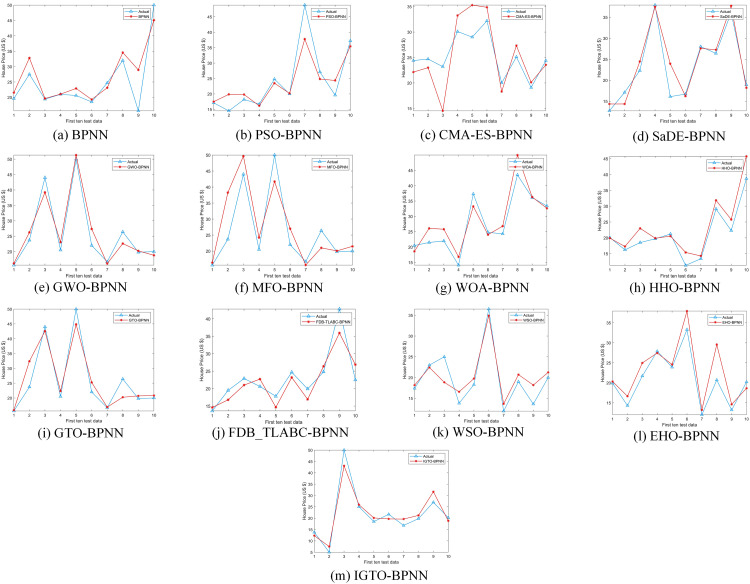
The prediction outcomes of SI-BP on Boston.

**Fig 9:**
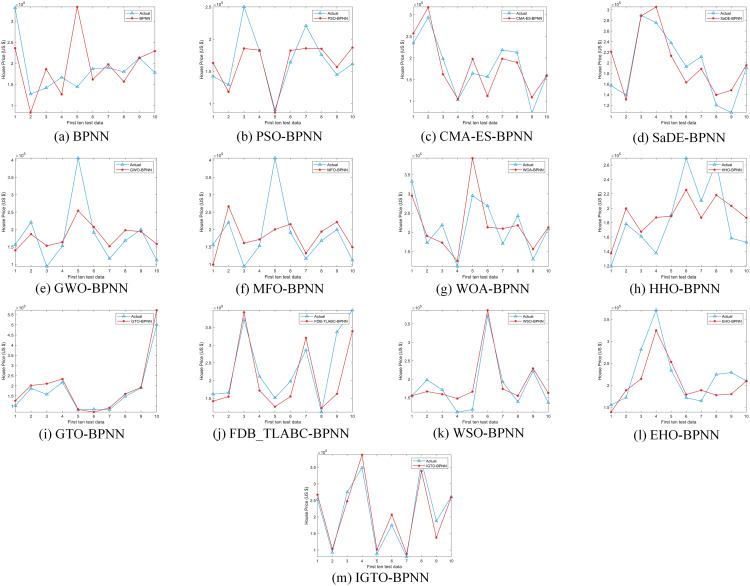
The prediction outcomes of SI-BP on Ca-Bay.

**Fig 10:**
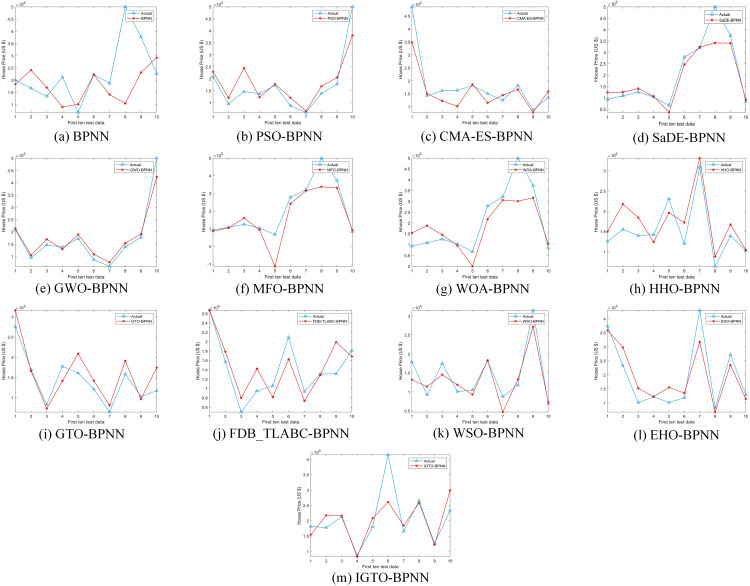
The prediction outcomes of SI-BP on Ca-Land.

**Fig 11:**
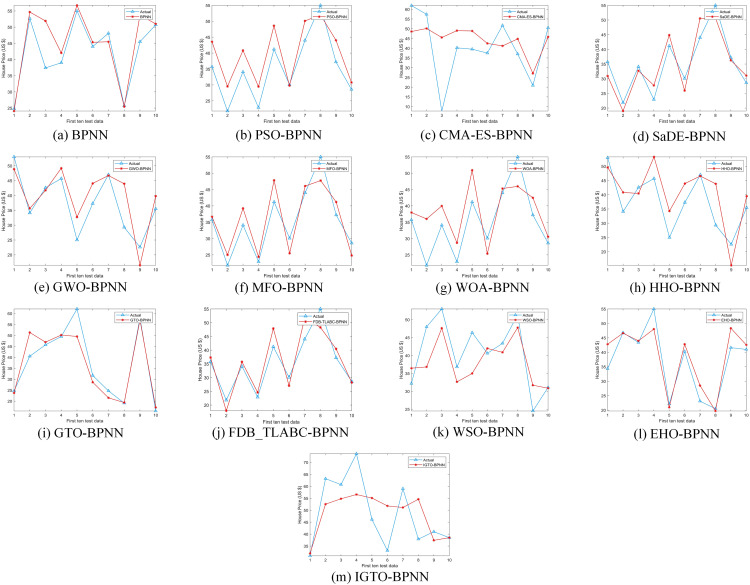
The prediction outcomes of SI-BP on Taiwan.

Upon comparing the prediction conclusions of the models across these datasets (only showing the first ten test data in Figs 8–11, it is apparent that the proposed IGTO-BPNN prediction model delivers the most favorable outcomes. Figs 8–11 show the validation results of the twelve SI-BPNN models and the original BPNN on four house price datasets. To improve readability, only the first 10 experimental results from the test data are shown. In the Boston and Ca-Bay datasets, the house prices predicted by IGTO-BPNN highly match the true values. Although the predicted values of IGTO-BPNN in the first ten test data in Ca-Land and Taiwan datasets have some deviation from the true values, IGTO-BPNN still achieves the best results in terms of evaluation metrics.

### 5.2. Qualitative analysis

#### 5.2.1. Exploration and exploitation.

In swarm intelligence algorithms, two phases, search and exploitation are crucial. In the search phase, individuals of the population need to traverse the solution set space as much as possible to find better solutions in the unknown space. The development phase, on the other hand, focuses on the known space and searches within the neighborhood of the current solution, aiming to find potential better solutions. Keeping a balance between the search and development phases can not only help the algorithm converge to the optimal solution quickly and improve the solution efficiency, but also allow the algorithm to show good adaptability and robustness when solving various types of optimization problems. Therefore, an excellent SI algorithm should achieve a good balance between the search and development phases. In this paper, the IGTO algorithm is used to optimize the weights and biases of the BP neural network, followed by the prediction of the four house price datasets. The calculation of the percentages of the search and development, respectively, using Eq. 26, as a way to assess how well the algorithm balances these two aspects.


$$\begin{array}{c} \phi =\frac{\text{1}}{D}\sum_{d=1}^{D}\frac{\text{1}}{Nall}\sum_{i=1}^{Nall}\left|median(\varsigma_{d})-\varsigma_{i,d}\right|\\ Exploration=\frac{\phi }{\phi_{max}}\times 100\%\\ Exploitation=\frac{\left|\phi -\phi_{max}\right|}{\phi_{max}}\times 100\% \end{array}$$ (26)

**Fig 12:**
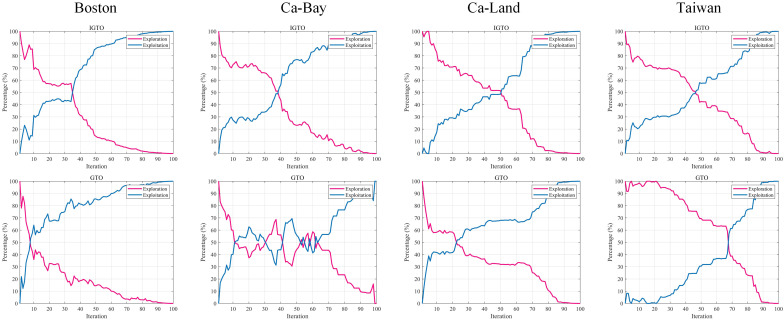
Exploration and exploitation.

In Eq. 26, *D* is the variable dimension of the optimization problem and *Nall* is the number of populations. $\varsigma_{i,d}$ is the d-th dimension of the i-th population individual. With Eq. 26, the percentage of the search and development phases of the IGTO algorithm is calculated, as shown in Fig 12.

Fig 12 visualizes the balance between the search and exploitation phases when the IGTO algorithm optimizes the weights and biases of the BP neural network. The intersection of search and exploitation in the IGTO algorithm is generally in the middle of the iteration. At the beginning of the iteration, the population requires a higher search ratio to fully explore the solution set space; then at the middle of the iteration, it gradually enters the exploitation phase. And in the late iteration, the development ratio of IGTO is higher, which helps to improve the convergence accuracy and solution speed of the optimization problem. It should be pointed out that the GTO algorithm has localized development at the beginning of iteration in Boston and Ca – Land datasets; in Ca – Bay dataset, it oscillates back and forth between the search and development phases, which is prone to waste of computational resources; and in Taiwan dataset, the search phase consumes too much time, and there is a problem of slow convergence. It can be seen that this paper adopts the fitness allocation strategy, the Cauchy perturbation strategy and the elite evolution strategy to improve the GTO algorithm, which is effective and achieves an even balance between the search and development phases.

#### 5.2.2. Ablation experiment.

This paper introduces three enhanced strategies to the GTO algorithm: (1) the fitness allocation strategy is employed to dynamically adjust the population positions, ensuring diversity among individuals; (2) the Cauchy variation strategy is applied to prevent the algorithm from converging to finite optimal solutions; (3) the elite evolution mechanism is introduced to evolve elite individuals and eliminate weaker ones, enhancing the algorithm’s global search capability. Ablation experiments are conducted to assess the impact of these three strategies on the performance of the IGTO-BPNN model. IGTO1-BPNN refers to using only strategy 1, IGTO2-BPNN refers to using only strategy 2, IGTO23-BPNN refers to using both strategy 2 and 3, and so on. Tables 9–12 present the comparison indices for each ablation experiment.

**Table 9:** The indexes of Ablation study on Boston.

Models	MAE	MAPE	R^2^	RMSE	SMAPE	SD	Time (s)
GTO-BPNN	2.6158	0.1239	0.8375	5.2865	0.1241	5.0159	110.5479
IGTO1-BPNN	2.6000	0.1358	0.8417	4.8056	0.1260	4.9242	97.8459
IGTO2-BPNN	2.5853	0.1248	0.8377	5.2347	0.1244	4.0604	93.1953
IGTO3-BPNN	2.5574	0.1209	0.8478	5.2575	0.1222	4.0446	88.6982
IGTO12-BPNN	2.5786	0.1210	0.8417	4.8103	0.1230	4.2657	87.5126
IGTO13-BPNN	2.5679	0.1220	0.8455	4.8110	0.1237	4.2034	93.1842
IGTO23-BPNN	2.4845	0.1189	0.8508	4.6981	0.1213	3.9114	89.4499
IGTO-BPNN	**2.2013**	**0.1145**	**0.8881**	**3.2023**	**0.1108**	**3.3624**	**83.4011**

**Table 10:** The indexes of Ablation study on Ca-Bay.

Models	MAE	MAPE	R^2^	RMSE	SMAPE	SD	Time (s)
GTO-BPNN	3.2949	0.1867	0.7131	5.1705	0.1727	5.5135	95.1052
IGTO1-BPNN	3.2724	0.1834	0.7133	4.5223	0.1739	4.4976	88.9970
IGTO2-BPNN	3.2871	0.1933	0.7215	4.1665	0.1797	4.8691	88.6910
IGTO3-BPNN	3.2817	0.1851	0.6822	5.8679	0.1758	4.9454	88.5140
IGTO12-BPNN	3.2406	0.1792	0.7204	4.9383	0.1747	4.3295	86.8189
IGTO13-BPNN	3.2302	0.1718	0.7191	4.8982	0.1682	4.1408	85.7749
IGTO23-BPNN	3.1586	0.1650	0.7129	4.2776	0.1615	4.1711	85.4811
IGTO-BPNN	**3.0292**	**0.1512**	**0.7346**	**3.3384**	**0.1514**	**4.0536**	**83.5672**

**Table 11:** The indexes of Ablation study on Ca-Land.

Models	MAE	MAPE	R^2^	RMSE	SMAPE	SD	Time (s)
GTO-BPNN	3.2360	0.1949	0.7881	5.7398	0.1877	4.6025	153.4851
IGTO1-BPNN	3.2638	0.1927	0.7846	5.3508	0.1870	4.5380	112.5489
IGTO2-BPNN	3.2179	0.1879	0.7856	5.1971	0.1883	4.6290	125.7489
IGTO3-BPNN	3.2880	0.1931	0.7909	5.3554	0.1874	4.3963	103.5423
IGTO12-BPNN	3.2465	0.1875	0.7915	4.2041	0.1868	4.5071	102.4712
IGTO13-BPNN	3.1075	0.1832	0.7924	4.6104	0.1861	4.1088	93.5781
IGTO23-BPNN	3.1465	0.1791	0.7963	3.8642	0.1854	4.0457	88.2158
IGTO-BPNN	**3.0759**	**0.1760**	**0.8075**	**3.3879**	**0.1848**	**3.9757**	**72.6272**

**Table 12:** The indexes of Ablation study on Taiwan.

Models	MAE	MAPE	R^2^	RMSE	SMAPE	SD	Time (s)
GTO-BPNN	5.8964	0.1511	0.5505	7.0243	0.1509	8.0427	119.6245
IGTO1-BPNN	5.7966	0.1514	0.5747	6.8038	0.1486	7.8778	108.5498
IGTO2-BPNN	5.6509	0.1523	0.5922	6.7186	0.1453	7.8069	112.2143
IGTO3-BPNN	5.8641	0.1504	0.6162	6.6425	0.1464	7.6970	100.3168
IGTO12-BPNN	5.7707	0.1451	0.5744	6.6461	0.1469	7.9180	90.5178
IGTO13-BPNN	5.8969	0.1466	0.5870	6.7088	0.1470	7.6855	85.7462
IGTO23-BPNN	5.4236	0.1460	0.6129	6.3824	0.1430	7.6927	88.6235
IGTO-BPNN	**5.2275**	**0.1434**	**0.6583**	**6.2697**	**0.1393**	**7.3309**	**75.1857**

On all four datasets (Boston, Ca-Bay, Ca-Land, and Taiwan), IGTO-BPNN shows excellent performance. Specifically, on the Boston dataset, IGTO-BPNN performs best in all metrics (MAE 2.2013, RMSE 3.2023, R^2^ 0.8881) under all strategies. On the Ca-Bay dataset, IGTO-BPNN and IGTO23-BPNN incorporating several enhancement strategies performed close to each other, with the former improving in R^2^. For the Ca-Land dataset, IGTO-BPNN performs better in terms of overall accuracy, especially in terms of SMAPE values, and the performance difference is not significant, although IGTO23-BPNN is able to slightly improve R^2^. On the other hand, on the Taiwan dataset, IGTO-BPNN performs better in MAE, RMSE, and SMAPE, showing its stability and accuracy, and the overall effect is not significantly different from that of IGTO-BPNN, despite the fact that IGTO23-BPNN improves R^2^. Meanwhile, the size of SD value can reflect the stability of the algorithms in the ablation experiments. The SD of IGTO-BPNN is always ranked first, which indicates that the three strategies enhance each other to optimize their performance. The SD of the remaining control group still fluctuates and the value is smaller than that of IGTO-BPNN. The running time can directly reflect the solving efficiency of the model. Compared with the original GTO-BPNN model, the method in this paper can both enhance the network performance and guarantee the solving speed. The table reveals the following observations:

1) Using only strategies ①, ②, or ③ results in a slight improvement in the IGTO-BPNN model’s performance.
2) Employing strategies ①② or ①③ together does not yield as significant an improvement as strategy ②③.
3) The combination of all three strategies, ①②③, leads to the best model performance, thus supporting the proposed IGTO-BPNN model.

**Fig 13:**
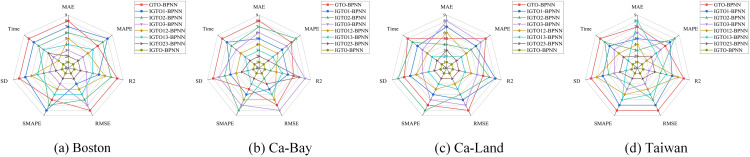
Ranking chart of evaluation metrics for ablation experiments.

Fig 13 shows the ranking graphs of the evaluation metrics in Tables 9–12, which shows the improvement effect of the three strategies on the GTO algorithm, with IGTO-BPNN having the best ranking.

### 5.3. Comparison with machine learning models

This section evaluates the performance of the IGTO-BPNN model in house price prediction by comparing it with other classical machine learning models. Four machine learning models, LSTM, ELM, RBF, and CNN, are used in the experiments. The evaluation indexes of different machine learning prediction models are reported in Tables 13–16. The CNN model continues to exhibit strong performance in both prediction results and evaluation metrics, surpassing the performance of BPNN optimized by other intelligent algorithms.

**Table 13:** Indexes of Machine learning models on Boston.

Models	MAE	MAPE	R^2^	RMSE	SMAPE	SD	Time (s)
LSTM	2.5723	0.1283	0.8226	5.7849	0.1224	3.0901	96.2005
ELM	2.8159	0.1426	0.8099	3.5870	0.1412	4.1919	119.6036
RBF	2.7089	0.1417	0.7914	3.7689	0.1363	3.8966	112.5898
CNN	2.2753	0.1160	0.8543	3.2614	**0.1104**	**2.9621**	146.5198
IGTO-BPNN	**2.2013**	**0.1145**	**0.8881**	**3.2023**	0.1108	3.3624	**83.4011**

**Table 14:** Indexes of Machine learning models on Ca-Bay.

Models	MAE	MAPE	R^2^	RMSE	SMAPE	SD	Time (s)
LSTM	3.4135	0.1970	0.6647	10.0236	0.1786	5.2933	97.1406
ELM	3.4974	0.1963	0.6067	5.7224	0.1810	4.5425	118.8470
RBF	3.4144	0.1937	0.6212	4.8052	0.1777	4.5457	130.7619
CNN	**2.8419**	0.1561	**0.7352**	3.5529	**0.1453**	**3.6050**	152.1012
IGTO-BPNN	3.0292	**0.1512**	0.7346	**3.3384**	0.1514	4.0536	**83.5672**

**Table 15:** Indexes of Machine learning models on Ca-Land.

Models	MAE	MAPE	R^2^	RMSE	SMAPE	SD	Time (s)
LSTM	3.1306	0.1942	0.7743	10.5919	0.1853	**3.8947**	214.5411
ELM	3.5720	0.2288	0.7070	5.2795	0.2163	6.4982	95.8420
RBF	3.5035	0.2256	0.7113	5.9408	0.2162	4.9668	148.4514
CNN	**3.0486**	0.1864	0.7784	4.0841	**0.1779**	4.4064	261.2017
IGTO-BPNN	3.0759	**0.1760**	**0.8075**	**3.3879**	0.1848	3.9757	**72.6272**

**Table 16:** Indexes of Machine learning models on Taiwan.

Models	MAE	MAPE	R^2^	RMSE	SMAPE	SD	Time (s)
LSTM	5.3397	0.1522	0.6386	8.2160	0.1434	**6.6422**	83.6615
ELM	5.3952	0.1608	0.6115	8.1999	0.1511	8.4270	186.2389
RBF	5.6028	0.1649	0.6087	9.5520	0.1566	7.6470	108.7473
CNN	5.2892	0.1592	0.6389	7.2885	0.1425	8.3090	113.1657
IGTO-BPNN	**5.2275**	**0.1434**	**0.6583**	**6.2697**	**0.1393**	7.3309	**75.1857**

**Fig 14:**
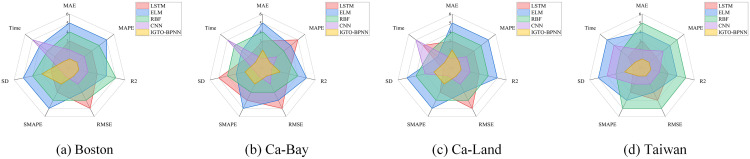
Ranking of machine Learning evaluation metrics.

Fig 14 shows the ranking result graphs in Tables 1316. Compared with the other four machine learning models, IGTO-BPNN gets 18 first places in the evaluation metrics, while CNN only gets 8 first places, which shows that IGTO-BPNN is competitive in house price prediction.

The IGTO-BPNN outperforms the other models in the Boston dataset (Table 13) in terms of MAE (2.2013), MAPE (0.1145), R^2^ (0.8881), and RMSE (3.2023), demonstrating excellent accuracy and stability. LSTM and CNN follow closely, though LSTM (MAE 2.5723, R^2^ 0.8226) and CNN (MAE 2.2753, R^2^ 0.8543) also perform well, but they are still slightly inferior to IGTO-BPNN in all the metrics. In the Ca-Bay dataset (Table 14), the MAPE (0.1512) and RMSE (3.3384) of IGTO-BPNN are lower than other models, and R^2^ (0.7346) is higher than most models, which demonstrates its excellent prediction performance. CNN model has the lowest MAE (2.8419), and R^2^ (0.7352) is not low but has no advantage over other models, and the overall performance is only second to IGTO-BPNN. In the Ca-Land dataset (Table 15), the advantage of IGTO-BPNN is obvious, and the advantage of RMSE (3.3879) is higher than other models. IGTO-BPNN has the best performance significantly better than LSTM, ELM, RBF and CNN models in terms of MAPE (0.1760) and R^2^(0.8075). The Taiwan dataset (Table 16), IGTO-BPNN dominates this dataset. Although the MAE (5.2275) of IGTO-BPNN is slightly higher, its RMSE (6.2697) and SMAPE (0.1393) are at the top of all the models and its R^2^ (0.6583) is slightly higher than the other models. The LSTM model has a MAE (5.3397), RMSE (8.2160) and SMAPE (0.1434) are on the high side, and the prediction effect is significantly less than that of IGTO-BPNN. When comparing IGTO-BPNN with CNN, (1) Although IGTO-BPNN achieves the highest comprehensive ranking, its indicators are only slightly better than CNN. For instance, in the Boston dataset, RMSE is 3.2023 for IGTO-BPNN and 3.2614 for CNN. (2) Moreover, CNN’s metrics are only slightly higher than those of IGTO-BPNN. For example, in the Ca-Land dataset, SMAPE is 0.1779 for CNN and 0.1848 for IGTO-BPNN. The IGTO-BPNN model demonstrates excellent performance in house price prediction and remains competitive when compared to classical machine learning models.

**Fig 15:**
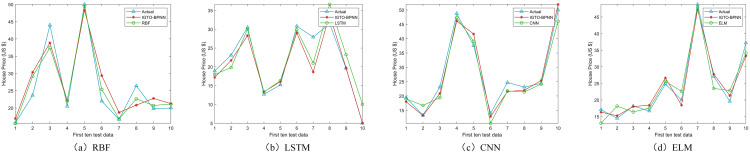
Results of machine learning models on Boston.

**Fig 16:**
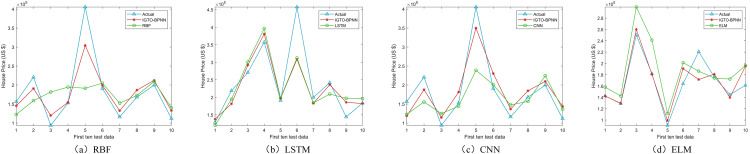
Results of machine learning models on Ca-Bay.

**Fig 17:**
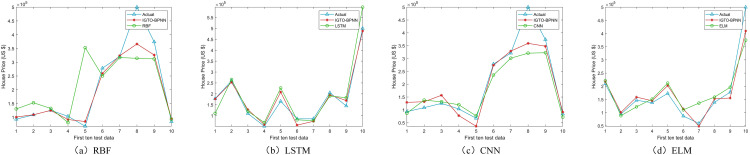
Results of machine learning models on Ca-Land.

**Fig 18:**
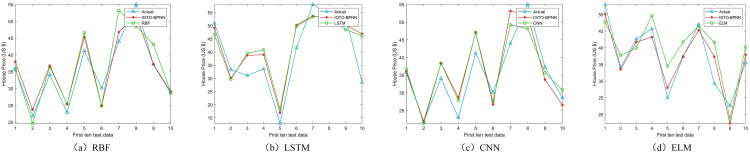
Results of machine learning models on Taiwan.

In the Boston and Ca-Bay datasets, CNN obtained the best SD twice; in the remaining datasets, LSTM performed optimally. In the four datasets, the IGTO-BPNN model did not obtain the optimal SD once, which suggests algorithmic volatility in the model. However, the IGTO-BPNN obtains four second-place finishes, and the gap with the optimal SD is always very small. CNN and LSTM for house price prediction require a large amount of runtime to obtain the optimal SD. In contrast, the method in this paper obtains a better prediction accuracy with less computational resources, which is rare. Figs 15–18 show the real values and prediction outcomes of every model (showing only the first ten test data points), demonstrating that the IGTO-BPNN model outperforms the other models in prediction accuracy.

## 6. Conclusion

This paper proposes a novel method for house price prediction, using an improved Artificial Gorilla Troops Optimizer (IGTO) to enhance the conventional BP neural network. By refining the initial weights and biases, the IGTO-BPNN model achieves more accurate and efficient house price predictions. To evaluate the performance of the IGTO-BPNN model, two sets of control experiments are conducted. The first compares the IGTO-BPNN model with BPNN optimized by eleven different SI algorithms, including PSO, CMA-ES, SaDE, GWO, MFO, WOA, HHO, GTO, FDB_TLABC, WSO, EHO. The second comparison is made against four classical machine learning models: LSTM, ELM, RBF, and CNN. All the above models show the performance of house price prediction through five indicators: MAE, MAPE, R^2^, RMSE, SMAPE. In this paper, the standard deviation and running time are also counted, and experimental validation is carried out using four house price datasets. The experimental results show that the IGTO-BPNN model not only ensures the algorithm stability, but also has a faster solving efficiency in the house price prediction problem. Compared with the traditional BP neural network, the model improves 17.66%, 18.27%, 28.10%, 49.35% and 24.83% in five evaluation indexes, which is sufficiently competitive with machine learning techniques.

The IGTO-BPNN model proposed in this study has certain advantages in the problem of house price prediction. Economically, it can accurately judge the trend of house price for investors, reduce the risk, and promote the stability of the market and the economy; socially, it can help the government to formulate a reasonable housing policy, safeguard people’s livelihood, and promote harmony; technically, the improved strategies and optimized algorithms can provide researchers with new ideas, which can also be extended to other prediction problems and promote the development of technology. However, this study still has some limitations. (1) Only four specific public datasets are selected for the experiment, which cannot comprehensively cover the house prices in different regions, economic environments and market conditions around the world, which limits the generalizability of the research results. (2) In the experimental process, the impact of the unique characteristics of different datasets on the model performance is not fully considered. For example, the datasets may have uneven data distribution and different ways of handling missing values. (3) The experimental comparison group contains 11 SI-BPNNs and four machine learning techniques, which fails to cover all the representative advanced algorithms and models in the field. Some emerging algorithms and models with better performance may not be included in the comparison, making it difficult to comprehensively assess the relative advantages of the IGTO – BPNN model among many algorithms.

Future work can be advanced in the following aspects: (1) Collecting more house price datasets from different regions, economic levels and market types to improve dataset diversity and enhance the model’s generalization ability and generalizability. (2) Analyze the features of different datasets in depth to understand their uniqueness, select features in a targeted manner, and optimize data preprocessing methods and model parameters. (3) Pay attention to the latest achievements in the field, and include more representative advanced algorithms for comparison, such as deep learning-based optimization algorithms and novel swarm intelligence algorithms.
